# Current Trends in Pediatric Migraine: Clinical Insights and Therapeutic Strategies

**DOI:** 10.3390/brainsci15030280

**Published:** 2025-03-06

**Authors:** Adnan Khan, Sufang Liu, Feng Tao

**Affiliations:** Department of Biomedical Sciences, Texas A&M University College of Dentistry, 3302 Gaston Ave., Dallas, TX 75246, USA; akhan92@tamu.edu (A.K.); sufang.liu@tamu.edu (S.L.)

**Keywords:** pediatric migraine, prodrome, aura, headache, psychiatric comorbidities, nutraceuticals

## Abstract

**Background/Objectives:** Pediatric migraine is a prevalent neurological disorder that significantly impacts children’s quality of life, academic performance, and social interactions. Unlike migraines in adults, pediatric migraines often present differently and involve unique underlying mechanisms, making diagnosis and treatment more complex. **Methods:** This review discusses the clinical phases of pediatric migraine, key trigger factors, sex- and age-related differences, and the role of childhood maltreatment in migraine development. We also discuss episodic syndromes such as cyclic vomiting syndrome, abdominal migraine, benign paroxysmal vertigo, and benign paroxysmal torticollis, along with comorbidities such as psychiatric disorders, sleep disturbances, and epilepsy. **Results:** The underlying pathophysiological mechanisms for pediatric migraines, including genetic predispositions, neuroinflammation, and gut microbiota dysbiosis, are summarized. Current therapeutic strategies, including conventional and emerging pharmacological treatments, nutraceuticals, and non-pharmacological approaches, are evaluated. Non-pharmacological strategies, particularly evidence-based lifestyle interventions such as stress management, diet, hydration, sleep, exercise, screen time moderation, and cognitive behavioral therapy, are highlighted as key components of migraine prevention and management. The long-term prognosis and follow-up of pediatric migraine patients are reviewed, emphasizing the importance of early diagnosis, and tailored multidisciplinary care to prevent chronic progression. **Conclusions:** Future research should focus on novel therapeutic targets and integrating gut–brain axis modulation, with a need for longitudinal studies to better understand the long-term course of pediatric migraine.

## 1. Introduction

Migraine is a neurological disorder characterized by moderate to severe throbbing and pulsating pain on one side of the head, nausea, vomiting, phonophobia, and photophobia [1,2]. This debilitating pain can last for several days and adversely affect the sufferer’s daily activities [3]. Globally, an estimated 1 billion people suffer from migraines, and this disorder remains the second leading cause of disability [4]. Migraines, as classified by the International Classification of Headache Disorders (ICHD)-3, include two main subtypes: migraine without aura and migraine with aura [5]. A migraine without aura, or common migraine, involves recurring headaches lasting 4 to 72 h, typically on one side, pulsating, and accompanied by nausea and sensitivity to light and sound [5,6]. Migraine with aura, or classical migraine, includes reversible visual, sensory, or neurological symptoms lasting about 15–20 min, occurring in 15–30% of cases, with visual auras in over 90% of these [5].

Children and adolescents are more prone to migraines than any other type of headache, which can significantly impair their school performance [7]. In contrast to migraines, which often present with symptoms like photophobia, phonophobia, nausea, and vomiting, tension-type headaches (TTHs) involve more continuous, band-like pain and lack these distinct features. Accurate diagnosis is crucial, as both conditions can affect a child’s daily life and require different treatment approaches [8]. About 11% of pediatric patients suffer from migraine headaches [9]. The ICHD-3 defines chronic migraine as headache attacks that occur at least 8 days per month for more than 3 months with the characteristics of migraine headaches [10]. In children and adolescents, chronic migraine is reported to affect 1% of the population [10]. A pediatric migraine not only affects daily academic activities, friendships, school performance, and family life but also has secondary psychological effects. Migraine in pediatrics can significantly hinder neurological development in children and adolescents, transforming it into a social problem if left untreated [11]. One of the biggest risks of childhood migraine is the potential for it to progress into chronic migraine, where occasional episodes become more frequent, leading to 15 or more migraine days per month for at least 3 months, resulting in increased disability [8]. A better diagnosis and treatment of pediatric migraines can reduce the prevalence and burden of pediatric migraines in adulthood [12].

This review aims to summarize recent research on pediatric migraine, focusing on its diagnostic criteria, clinical features, episodic syndrome, comorbidities, and underlying mechanisms, including genetic factors, neuroinflammation, sex differences, triggers, childhood maltreatment, and the gut–brain axis. We also evaluate current treatments, including pharmacological, nutraceutical, and non-pharmacological approaches, providing insights to guide future research and the development of tailored treatment strategies for pediatric migraine.

After providing an overview and outlining the aim of the review, the following section delves into the ICHD-3 diagnostic criteria, highlighting the key differences and similarities between adult and pediatric migraine diagnoses.

## 2. ICHD-3 Migraine Diagnostic Criteria: Adults vs. Children

Pediatric and adolescent migraines differ from adult presentations, often manifesting as more frequent bilateral headaches with shorter duration [13,14]. The ICHD-3 provides specific criteria for diagnosing migraine in adults and children, acknowledging key differences between the two groups [15,16]. For adults, a migraine diagnosis requires at least five attacks that meet the following criteria: the headache lasts between 4 and 72 h, and it includes at least two of the following characteristics: unilateral location, pulsatile quality, moderate-to-severe intensity, or aggravation by routine physical activity. Additionally, at least one of the following symptoms must be present: nausea and vomiting, or photophobia and phonophobia [15,16]. In children, the criteria are slightly modified to account for developmental differences. The headache can be shorter, lasting between 2 and 72 h. The pain may be either unilateral or bilateral, with most children reporting bilateral pain. Like adults, the headache must include at least two characteristics: pulsatile quality, moderate-to-severe intensity, or aggravation by routine physical activity. The presence of nausea, vomiting, photophobia, or phonophobia is also required. However, photophobia and phonophobia in children are often inferred from behavior, such as seeking out a dark and quiet room, rather than being verbally reported [15,16]. Furthermore, children with migraines often experience a broader spectrum of gastrointestinal symptoms, including abdominal pain, nausea, vomiting, diarrhea, and constipation compared to adults [17]. The characteristics of pediatric and adult migraines are summarized in Table 1.

## 3. Sex- and Age-Specific Differences in Childhood Migraine

Numerous studies have been done in adulthood on sex differences in migraine, but fewer have been conducted in childhood and adolescence [18,19,20,21]. Women aged 18–44 experience migraines twice more than men in terms of frequency, intensity, and duration [18,19]. The higher prevalence of migraines in females during reproductive years, compared to pre-puberty and post-menopause, indicates a significant role of hormonal factors in migraine pathogenesis [20]. In childhood, migraine prevalence increases with age, typically affecting girls aged 8–14 and boys aged 9–15. Interestingly, migraine prevalence peaks between ages 35 and 45, with the female-to-male ratio increasing from 2:1 at age 20 to 3.3:1 at age 40 [19]. Among pre-pubertal children, migraines are relatively rare (3–10%) without significant sex differences, and episodic symptoms like benign paroxysmal torticollis and abdominal migraines might be due to more mature brainstem effectors. However, the landscape shifts after puberty. The hypothalamus regulates autonomic systems, increasing female migraine sensitivity [21]. The changes in the neuroendocrine axis with age lead to post-menopausal migraine improvement due to low estrogen and high follicle-stimulating hormone (FSH) levels [21]. In summary, pre-pubertal boys have a higher migraine prevalence, which shifts to a higher prevalence in post-pubertal girls.

In addition to sex differences in pediatric migraine, age is another crucial factor shaping its clinical presentation. Recent research indicates that pediatric migraine varies significantly by age. In pre-school children, migraine typically presents with shorter durations and atypical symptoms, likely due to challenges in self-reporting, while school-age children and adolescents exhibit more classic features and a higher prevalence of the disorder [22]. Likewise, a school-based study found that 10.4% of children had migraine (1.7% chronic, 8.6% episodic), with a higher prevalence in girls. By age 12, chronic migraine frequency doubled, with older age, female gender, and family history as key risk factors; in adolescents, migraine prevalence increased to 18.6% (1.5% chronic, 17.1% episodic), and twice as many chronic cases became headache-free by puberty. These results highlighted that the prevalence of both episodic and chronic migraine rises with age, a trend that is particularly evident among older children and adolescents [23]. These age-related variations highlight the importance of tailoring diagnostics and treatment strategies to each developmental stage.

## 4. Clinical Manifestations in Pediatric Migraine: Triggers, Phases, and Variants

The clinical manifestations of pediatric migraines vary with age: infants may display discomfort through “head banging”, toddlers often present with vomiting and abdominal pain, and as children grow older, migraines typically become more prolonged and intense, with symptoms including lethargy, nausea, vomiting, bifrontal, bitemporal, or retroorbital headache, photophobia, phonophobia, nasal congestion, dehydration, edema, and diarrhea [17,24]. The following subsections provide an in-depth look at triggers, the phases of migraine attacks, and the various migraine variants observed in children, which are critical for accurate diagnosis and management.

### 4.1. Trigger Factors in Pediatric Migraine

Patients with migraines often identify certain factors that increase the likelihood of an attack. These “trigger factors” are defined as specific internal or external events or exposures that increase the risk of migraines within a short period [25]. Trigger categories include behavioral, environmental, infectious, dietary, chemical, and hormonal factors, suggesting a hyperexcitable brain that reacts quickly to various stimuli. Identifying and avoiding triggers is key to managing migraines [26]. The most frequently reported migraine triggers in children include sleep deprivation, stress, warm climate, noise, and bright lights [26,27]. Among these, stress is the leading trigger for pediatric migraines, with home and school environments contributing to attacks. Stress not only disrupts brain function by altering gene expression but also increases susceptibility to the development of disorders like depression and anxiety [28]. In most cases, the time between trigger exposure and migraine onset in children is often less than 3 h [26]. Additionally, foods such as chocolate, caffeine, milk, and cheese are frequent migraine triggers [28]. Dietary habits play a crucial role in triggering headaches in children and adolescents with migraines, possibly impacting the migraine process by affecting serotonin and norepinephrine levels, causing changes in blood vessels, or activating critical brain pathways [28]. This connection underscores the importance of nutrition in managing migraines in children and adolescents. It is widely believed that altered neurovascular homeostasis disrupts hypothalamic networks, leading to pain and other symptoms in a genetically sensitive brain [29]. The hypothalamus, vital for both homeostasis and pain regulation, plays a crucial role in triggering and advancing migraines [30].

### 4.2. Migraine Phases in Children

The American Migraine Foundation outlines four distinct phases of a migraine attack: prodrome, aura, headache, and postdrome, each with specific symptoms, though symptoms can overlap between phases [31].

#### 4.2.1. Prodrome Phase (Pre-Headache Phase)

As defined by the ICHD-3, prodromal symptoms appear up to 48 h before the headache phase in migraine without aura or before the aura phase in migraine with aura [15,16]. The key premonitory symptoms reported in adults and children include cognitive and mood changes (concentration difficulties, memory complaints, confusion, irritability), homeostatic and hormonal shifts (food cravings, thirst, sleep disruption, yawning), sensory sensitivities (mild head discomfort, light/sound/smell sensitivity, neck pain, allodynia, nausea), and cranial autonomic signs (tearing, nasal congestion, runny nose, ear fullness, abnormal taste, throat swelling) [32]. Premonitory or prodromal symptoms are believed to affect over two-thirds of children, with the most frequently reported symptoms including fatigue, irritability, facial pallor, and dark circles around the eyes [33]. In the premonitory phase, the hypothalamus triggers early signs like mood changes, fatigue, yawning, and food cravings [34]. These symptoms are linked to neurotransmitters such as orexins, neuropeptide Y, and dopamine, produced by hypothalamic neurons [34]. Experimental studies show that these neurotransmitters modulate pain transmission in the trigeminocervical complex, and neuroimaging confirms hypothalamic activation during this phase [34]. Further research is needed to verify whether premonitory symptoms stem from hypothalamic dysfunction or broader neural mechanisms and whether they constitute a distinct migraine phase [34].

#### 4.2.2. Aura Phase

The ICHD-3 defines aura as a cluster of neurological symptoms that persist for 5–60 min and are usually followed by or occur within 60 min of a headache [16]. Diagnosis requires at least two attacks with fully reversible symptoms (visual, sensory, speech/language, motor, brainstem, or retinal) and at least three of the following: gradual spread of one symptom over ≥5 min, two or more symptoms in succession, each symptom lasting 5–60 min, at least one unilateral symptom, at least one positive symptom, and aura accompanied by or followed within 60 min by headache [6]. These criteria apply to both adult and pediatric patients. According to ICHD-3, migraine with aura is classified into typical aura, brainstem aura, hemiplegic migraine (familial and sporadic), and retinal migraine. Hemiplegic and brainstem auras are considered more severe and can affect treatment decisions [16,35].

Approximately one-third of children and adolescents with migraine experience migraine with aura [35]. In children with migraine, visual aura is most common, followed by somatosensory and language-based symptoms [36]. Visual disturbances in children with migraine may include blurred vision, zigzag lines, scotomata, scintillations, black dots, kaleidoscopic patterns, and size distortions like micropsia or macropsia [37]. Other auras involve sensory symptoms (numbness or tingling), speech or language deficits (aphasia, dysarthria), motor deficits (hemiparesis), and brainstem symptoms such as vertigo, tinnitus, and diplopia [37]. Aura symptoms can vary significantly in a single episode or between attacks [37]. Due to challenges in verbal description, the onset, and characteristics of aura in young children remain uncertain. Children’s drawings effectively differentiate migraines from other headaches and convey auras and symptoms nonverbally [33]. The physiological basis of an aura is believed to be cortical spreading depression (CSD), an electrophysiological event characterized by a wave of depolarization followed by hyperpolarization that slowly propagates across the cerebral cortex [38]. As the wave passes over specific cortical areas, corresponding neurological symptoms arise and then subside [38]. CSD disrupts ionic balance, neurotransmitter levels, and brain blood flow in migraine [39]. Several mechanisms have been proposed to explain how CSD leads to the headache phase of migraine with aura (MA), including CSD initiating an inflammatory cascade, releasing nociceptive substances, and directly activating the trigeminocervical complex [35].

Migraine with brainstem aura (MBA), formerly known as basilar migraine, is reported as the most prevalent subtype of migraine in certain pediatric groups [37,40,41]. According to ICHD-3, MBA is diagnosed when a patient experiences aura without motor or retinal symptoms, along with at least two reversible brainstem-related symptoms such as dysarthria, vertigo, tinnitus, hypoacusis, diplopia, ataxia, or altered consciousness (Glasgow Coma Score ≤ 13) [42]. The pathophysiology of MBA is not fully understood, with hypotheses including vasomotor dysfunction, cortical spreading depression, and neurogenic inflammation [43]. In MBA with disorders of consciousness (DOC), abnormal neurotransmitter activity, particularly increased GABA levels, may disrupt the reticular activating system (RAS), affecting consciousness [43]. The hypothalamus also plays a role, with decreased blood flow and connectivity in migraine pathways before an attack [44]. Abnormal cortical function may trigger aura symptoms and consciousness changes [45]. Managing MBA with DOC involves avoiding triggers like emotional stress, sleep disturbances, weather changes, and fatigue to reduce attack frequency [43].

Motor aura, characterized by motor weakness, is classified as a type of hemiplegic migraine (HM) [35]. HM is a clinically and genetically diverse disorder characterized by headache and motor weakness, often accompanied by visual, sensory, or speech aura, as well as impaired consciousness, cerebellar ataxia, and intellectual disability [46]. According to the ICHD-3, motor symptoms in hemiplegic migraine usually last less than 72 h, but in some cases, they can extend for several weeks [6]. HM can be sporadic (SHM) or familial (FHM) with autosomal dominant inheritance, and shares similarities in epidemiology, triggers, and clinical features but differs in onset age, genetics, and neurological profile [16,47]. HM is a rare, likely underdiagnosed condition that can appear in early childhood [48]. Gene mutations in CACNA1A, ATP1A2, and SCN1A are associated with FHM types 1, 2, and 3. Younger age of onset (under 10–16 years), prolonged motor aura, severe attacks, and triggers like mild head trauma, along with progressive ataxia or intellectual disability, are strong indicators of FHM with a specific mutation, compared to the sporadic form [49]. Its pathophysiology resembles typical migraine with aura but is more severe [46]. Pediatric HM differs from adult HM in features and gender distribution, necessitating tailored ICHD criteria [48]. SHM in children presents with longer, more severe but less frequent attacks compared to FHM, particularly early on [48]. Despite severe attacks, HM generally has a good prognosis, but ongoing follow-up is needed due to potential neurological comorbidities [47].

Retinal migraine is a rare cause of transient monocular aura and is recognized as a migraine subtype according to the ICHD-3 [50]. Migraine with retinal aura involves recurring reversible monocular visual symptoms, positive or negative, following an aura time-course and accompanied by headache [51]. The reversible monocular visual disturbances include scintillations, scotomata, or transient blindness, which are confirmed by clinical visual field exams or patient drawings during an attack [6]. Retinal migraine is rare in pediatric patients, comprising less than 2% of cases [35]. Patients with RM, particularly children, may struggle to differentiate between monocular visual loss and visual loss in the same hemifield of both eyes, making RM diagnosis more challenging [50]. Pediatric physicians must carefully interview patients to distinguish between monocular and unilateral visual signs during visual auras [50]. The different types of auras in pediatric migraines, along with their key symptoms and underlying mechanisms, are summarized in Table 2.

#### 4.2.3. Headache Phase

During the headache phase in children, the pain is typically described as pounding or throbbing and is often localized to the frontal-temporal areas, sometimes bilaterally [52]. Unlike adults, children rarely report occipital or neck pain during migraines [52]. The trigeminovascular system is considered the key mechanism for migraine headaches [53]. It involves first-order neurons in the trigeminal ganglion that innervate pain-sensitive intracranial structures, second-order neurons in the trigeminal nucleus caudalis that relay signals to the brainstem and thalamus, and third-order neurons in the thalamus that project to the somatosensory cortex, insula, and visual cortex [53]. Afferent projections from the trigeminal ganglion converge with inputs from the dorsal horn of the upper cervical spinal cord (C1-C2) in the trigeminal cervical complex (TCC), leading to referred pain in the periorbital, occipital, and neck regions [54]. Ascending pathways from the TCC transmit signals to the brainstem, thalamic, and cortical areas, contributing to symptoms like photophobia, phonophobia, and cognitive dysfunction during attacks [54]. Photophobia and phonophobia are key symptoms of migraine diagnosis, with up to 80% of children reporting them [8]. Migraine-associated photophobia involves optic nerve signals converging on trigeminovascular neurons, affecting the somatosensory cortex (exacerbating pain) or the visual cortex (increasing light sensitivity) via the retino-thalamo-cortical pathway [8]. Furthermore, nausea and vomiting commonly occur during the headache phase in younger children, likely due to vagal activation [55]. The higher frequency in young children suggests a link with cyclic vomiting syndrome [56].

##### Associated Sensory and Autonomic Features in Pediatric Migraine

Beyond pain, the headache phase frequently involves cranial autonomic symptoms (CASs), allodynia, and osmophobia, highlighting interactions between the trigeminal, autonomic, and sensory systems [57]. CASs, once considered characteristic of trigeminal autonomic cephalalgias, are now increasingly recognized in pediatric and adult migraines, likely due to trigeminal–autonomic reflex activation [57]. These symptoms include lacrimation, conjunctival injection, nasal congestion, rhinorrhea, forehead sweating, eyelid edema, ptosis, and miosis [58,59,60]. CASs are associated with higher attack frequency, greater severity, and increased risk of chronic migraine [61,62]. Unlike in adults, where CASs are typically unilateral, pediatric cases frequently present bilaterally, with a reported prevalence ranging from 40% to 68%, particularly in younger children [60]. Furthermore, in pediatric migraine, CASs do not exhibit the strong female predominance seen in adults, likely due to the minimal hormonal influence before puberty [62].

In addition to autonomic involvement, central sensitization is a key mechanism in migraine pathophysiology, resulting in allodynia, where normally non-noxious stimuli, such as light touch, combing hair, or wearing eyeglasses evoke pain [57,63]. Allodynia, driven by persistent trigeminothalamic activation, increases pain sensitivity and accelerates migraine progression [57,64,65]. Children with allodynia tend to have more severe, frequent attacks and a reduced response to acute treatments, suggesting a distinct migraine subtype [57]. Beyond allodynia, osmophobia (increased sensitivity to odors) is another often overlooked sensory symptom that may also reflect underlying central sensitization in pediatric migraine. Osmophobia, affecting up to 60% of pediatric migraineurs, is thought to stem from increased excitability in the olfactory-limbic system, which further exacerbates the sensory disturbances during an attack [66,67]. Research indicates that osmophobia is linked with more frequent migraine attacks, increased disability, and an overall intensified dysfunction in sensory processing, suggesting that it could mark a more severe migraine phenotype [57]. Additionally, the occurrence of osmophobia in children can serve as a distinguishing factor between migraines and TTH, paving the way for more precise diagnoses and the implementation of tailored management strategies [68,69].

#### 4.2.4. Postdrome Phase

The postdrome phase of a migraine, occurring after headache symptoms subside, often involves neuropsychiatric, sensory, and gastrointestinal symptoms [70]. Neck stiffness, difficulty concentrating, fatigue, light sensitivity, irritability, and nausea are the most typically reported postdrome symptoms [71]. Although these are sometimes attributed to migraine medications, no connection has been found between postdrome symptoms and the type of medication used [70]. In the pediatric population, 82% of patients report experiencing postdrome symptoms, with an average of 2.6 symptoms per migraine attack [72]. After the headache resolves, up to 90% of children with migraine report lingering symptoms such as fatigue, dizziness, difficulty concentrating, pallor, anorexia, and mood changes, with the return to baseline often taking two or more days, adding to the burden of the disease [8]. The postdrome phase of migraine involves locus coeruleus activation and cortical spreading depression, leading to reduced blood flow and suppressed brain activity [71]. Functional imaging studies highlight these mechanisms, emphasizing the need for better understanding due to their significant impact on recovery [71]. The pathophysiological mechanisms and clinical manifestations of the various migraine phases in children are summarized in Table 3.

### 4.3. Episodic Syndromes of Childhood Linked to Migraine

Migraine variants, officially termed episodic syndromes associated with migraine by the ICHD-3, are recurrent, periodic, and paroxysmal disorders seen in children and adolescents [73]. The ICHD-3 recognizes episodic syndromes in childhood that are linked to a higher risk of developing migraine [74]. Episodic syndromes in children include cyclic vomiting syndrome, abdominal migraine, benign paroxysmal vertigo, and benign paroxysmal torticollis [13,37,74]. Additionally, the ICHD-3 appendix lists infantile colic and alternating hemiplegia of childhood as syndromes potentially linked to migraine [74]. Many children with these syndromes may eventually develop typical migraine characteristics as they grow older [16]. Episodic migraines can have residual effects lasting up to 72 h, during which children may exhibit altered physical states, ranging from elation and increased energy to lethargy and exhaustion [17,37]. The clinical characteristics of these conditions are outlined in Table 4. Following the discussion of clinical manifestations, the next section focuses on the psychiatric and neurological comorbidities frequently accompanying pediatric migraines.

#### Vestibular Migraine in Pediatrics

Vestibular migraine and benign paroxysmal vertigo are the leading causes of vertigo in children and adolescents [75]. Vestibular migraine, the most common cause of balance disorders in children across all age groups, often with a family history of migraine, can be effectively diagnosed using the video head impulse test [76]. Various terms have been used to describe the coexistence of vestibular symptoms and migraine, including migraine-associated dizziness, migraine-associated vertigo, migrainous vertigo, migraine-related vestibulopathy, and benign paroxysmal vertigo [77]. First described by Kayan and Hood in 1984, a syndrome resembling vestibular migraine was noted in Greek antiquity. Dieterich and Brandt later coined the term “vestibular migraine” in 1999, now used for migraine-related vestibular symptoms [75]. Diagnosing vertigo in children and adolescents is a complex challenge, significantly impacting the quality of life and clinical outcomes [78]. The committee for the classification of vestibular disorders of the Bárány Society and the migraine classification subgroup of the International Headache Society (2021) introduced structured classifications to improve diagnostic accuracy: vestibular migraine of childhood (VMC), probable vestibular migraine of childhood (pVMC), and recurrent vertigo of childhood (RVC) [78,79]. VMC is the most migraine-associated category, requiring at least five episodes of moderate-to-severe vestibular symptoms lasting 5 min to 72 h, with a confirmed migraine history or at least half of the episodes featuring migraine-related symptoms such as headache, photophobia, or phonophobia [79]. pVMC shares similar characteristics but requires only three episodes and does not fully meet the strict VMC criteria. In contrast, RVC is defined by recurrent vertigo episodes lasting 1 to 72 h, but it lacks a clear migraine association, as neither a migraine history nor migraine-related features are present in more than half of the episodes [79]. These classifications help distinguish migraine-related vertigo from other vestibular disorders, ensuring more accurate diagnoses and tailored treatment strategies for pediatric patients.

## 5. Psychiatric and Neurological Comorbidities in Pediatric Migraine

Pediatric migraines are often comorbid with psychiatric and neurological conditions, including depression, anxiety, seizures, and sleep disturbances, which may contribute to long-term mental and physical health complications in adulthood [12,80,81,82,83]. The following subsections explore key psychiatric and neurological comorbidities in pediatric migraine, including sleep disorders, anxiety and depression, and epilepsy.

### 5.1. Sleep Disorders and Pediatric Migraine

The connection between sleep disturbances and headaches in children is bidirectional [12]. Sleep disorders (SDs) affect about 25% of children, with higher rates in those with other medical or neurological conditions [84]. In some areas, up to 80% of children experience sleep issues, leading to significant disability [85]. Among adolescents with headaches, up to 50% report insomnia symptoms, which are linked to more frequent and severe headache episodes [12]. Sleep deprivation and poor sleep habits greatly increase the risk of mental and physical health issues, accidents, and poor academic performance [85]. Poor sleep, whether too short, too long, or low quality, can trigger migraines and is associated with the chronicization of the condition [84]. Sleep disorders, common in children with migraines, include parasomnias, sleep apnea, and movement disorders [85]. Children with migraines may also experience excessive daytime sleepiness, potentially due to mood or medical disorders, poor sleep quality, medications, or increased headache severity [86,87]. Shared underlying pathophysiological processes may explain the complex, bidirectional relationship between headaches and sleep [12]. Sleep pathways, including circadian rhythm and rapid eye movement (REM)/non-REM activation, closely align with migraine regulation, involving neurochemicals like adenosine, melatonin, orexin, and calcitonin gene-related-peptide (CGRP) [85]. Recent evidence supports the theory that common brain structures and pathways, including the hypothalamus, raphe nuclei, and serotoninergic system, are involved in both sleep and migraine regulation [88].

### 5.2. Anxiety and Depression in Children with Migraine

Migraine is strongly associated with psychiatric disorders, especially anxiety and depression, which are the most common [89]. Migraine and depression are bidirectionally linked, with depression three times more common in migraine patients with a higher risk of developing depression [90]. A recent systematic review and meta-analysis of 80 studies published in JAMA Pediatrics found that children with migraines are more likely to experience anxiety and depression symptoms compared to those without migraines [81]. Decreased thalamic activity in migraines with comorbid depression suggests a habituation deficit involving noradrenergic, dopaminergic, and serotonergic pathways [91]. Studies using magnetic resonance spectroscopy have shown thalamocortical dysfunction in chronic migraines, with serotonin and other neurotransmitters contributing to migraine–depression comorbidity [91,92]. Additionally, stress, primarily regulated by corticotropin-releasing factor-containing neural circuits and the hypothalamic–pituitary–adrenal axis, can trigger depression in migraineurs [91]. Future studies should focus on distinguishing symptoms of mood disorders from those of pediatric migraine, as this distinction will enhance clinical practice, particularly in improving screening and treatment strategies for these comorbid conditions [92].

### 5.3. Comorbidity of Migraine and Epilepsy in Pediatric Patients

Migraine and epilepsy are common pediatric neurological disorders characterized by recurrent nervous system dysfunction, influenced by both genetic and environmental factors [93]. Migraine and epilepsy both result from cortical neuron hyperexcitability, with epilepsy involving synchronized discharges and migraine linked to cortical spreading depression [94]. They share overlapping symptoms, including sensorimotor and cognitive impairments, consciousness disturbances, visual issues, dizziness, paresthesia, hemiparesis, and aphasia [95]. The cellular link between migraine and epilepsy involves channelopathies, ion imbalances in neurons and glial cells, GABAergic and glutamatergic systems disruptions, and mitochondrial dysfunction [93]. Several mutations causing monogenic forms of epilepsy and migraine share genes involved in ion channel function. Mutations in CACNA1A, ATP1A2, and SCN1A, responsible for familial hemiplegic migraine types 1, 2, and 3 (FHM1, FHM2, and FHM3), are also linked to epilepsy [96]. These genes regulate ion channels, and their mutations lead to neuron hyperexcitability. CACNA1A encodes the alpha-subunit of voltage-gated P/Q calcium channels, while SCN1A encodes the alpha-subunit of sodium channel Nav1.1, associated with severe myoclonic epilepsy in infants [96]. Mutations in proline-rich transmembrane protein 2 (PRRT2), which affects neurotransmitter release, are linked to both migraine and benign familial infantile seizures [97,98].

## 6. Etiological Factors in Pediatric Migraine

Pediatric migraines are multifactorial, with risk factors including genetic predisposition, environmental triggers, and comorbid gastrointestinal disorders [17].

### 6.1. Genetic Factors in Pediatric Migraine

Pediatric migraines often have a genetic predisposition that can be triggered by environmental or physiological factors (drug exposure, diet, and stress) [99]. There is a genetic basis for certain migraines, including hemiplegic migraine, which include mutations in genes like CACNA1A (calcium channel), ATP1A2 (Na/K-ATPase), and SCN1A (sodium channel) [99]. These genes code for key components involved in ion channel function, including the α1 subunit of the P/Q-type voltage-gated calcium channel, the α2 isoform of the Na/K-ATPase, and the α1 subunit of the voltage-gated sodium channel, respectively [100]. Mutations in ATP1A, encoding a sodium-potassium ATPase subunit, have been found in individuals with both FHM type 2 and epilepsy. Likewise, mutations in SCN1A, which codes for a voltage-gated sodium channel subunit, are associated with FHM type 3 and epilepsy [101]. A meta-analysis of 22 studies identified 44 SNPs across 38 genomic loci associated with migraine, many of which are linked to genes expressed in vascular and gastrointestinal smooth muscle, shedding light on gastrointestinal symptoms in conditions like abdominal migraine and cyclic vomiting syndrome [102,103]. Additionally, migraine-related pathways, including serotonin, dopamine, and estrogen receptor systems, have been investigated [19]. Variants in the serotonin transporter gene and C677T polymorphisms show sex-specific links to migraine, with a stronger association in women. While links between estrogen receptor genes (ESR1, ESR2) and migraine have been suggested, larger-scale studies have yet to fully validate these associations [19].

### 6.2. Childhood Maltreatment and Pediatric Migraine

Childhood maltreatment has been linked to a higher risk of developing migraines [104]. The World Health Organization (WHO) divides maltreatment of children into four primary categories: physical abuse, emotional abuse, sexual abuse, and neglect [1]. Childhood maltreatment, including physical, sexual, and emotional abuse has been linked to headaches across all ages including children, adolescents, and adults [105]. Childhood maltreatment can alter neurobiological properties, disrupt neurodevelopment, and play a crucial role in the development of pain and psychopathology, which may emerge at any life stage [106]. Child maltreatment is a widespread global issue with lasting effects on physical and mental health, extending into adulthood [104]. This maltreatment causes limbic system changes, like those seen in migraines, affecting cognitive functions like memory, learning, and attention in children [105].

According to a meta-analysis, the prevalence of child abuse worldwide ranged from 1.0% to 70.1% depending on the location [107]. Physical domestic and familial violence affects about half of children from West Asia and Africa and one-third of children from South Asia [107]. In the United States, an estimated 38.1% of children aged 14 to 17 have experienced childhood abuse, including 18.1% physical abuse and 23.9% emotional abuse, while 64% of college students in China reported childhood abuse [104]. There is a strong link between physical and psychological factors in links between stress, migraines, and mental health conditions in children. Children often express their emotional distress or psychological issues as physical symptoms instead of directly expressing them [108]. Childhood maltreatment may cause headaches by altering the hypothalamic–pituitary–adrenal axis and interfering with the endocannabinoid, monoaminergic, oxytocinergic, and inflammatory systems [105]. Ultimately, childhood maltreatment is a significant risk factor for pediatric migraines and may affect individuals of all ages, likely due to its lasting impact on pain perception and stress response.

### 6.3. Role of Gut Microbiota in Pediatric Migraine

Recent research has shown that the diversity and abundance of the gut microbiome in children are associated with the occurrence of migraines, suggesting that gut microbiota could be potentially involved in the biological mechanisms of migraines [17]. The gut microbiota, a dynamic community of bacteria residing in the gastrointestinal (GI) tract, is integral to gut development, immune system differentiation, and the overall physical and mental health of the child [109]. The collection of bacteria, archaea, and eukarya inhabiting the GI tract is known as the ’gut microbiota’ which has co-evolved with the host over thousands of years to establish a complex, mutually beneficial relationship [110]. The gut microbiome is the collection of all microbial genomes and their functional products (metabolites) in the GI tract [111]. The GI tract is estimated to contain over 10^14^ microorganisms, consisting of approximately ten times more bacterial cells than human cells and more than 100 times the genomic content of the human genome [110]. Recent advances highlight the critical role of the gut microbiome in human health and disease [112]. The microbiome–gut–brain axis is a bidirectional communication network linking the gut and brain, crucial for understanding gut–brain interactions. It includes the gastrointestinal immune system, enteric neuroendocrine system, enteric nervous system, central nervous system (CNS), and the gut microbiome [111]. The microbiota provide numerous benefits to the host, such as enhancing gut integrity, shaping the intestinal epithelium, harvesting energy, protecting against pathogens, and regulating immunity. However, these functions can be disrupted by dysbiosis or altered microbial composition, which could contribute to the development of migraines [110].

Dysbiosis, characterized by reduced biological diversity or altered bacterial taxa abundance, can increase susceptibility to various diseases, including migraines [17]. Dysbiosis of the gut microbiome has been implicated in several neurological disorders, including migraine, depression, anxiety, autism spectrum disorder, Huntington’s disease, multiple sclerosis, stroke, amyotrophic lateral Sclerosis, Parkinson’s disease, Alzheimer’s disease, epilepsy, and neuropathic pain [113,114,115,116]. Multiple studies highlight the critical role of gut microbiota in the onset and persistence of migraines [117,118,119,120]. The gut microbiome composition significantly influences migraine susceptibility through mechanisms such as neuroinflammation, gut–brain signaling, and metabolic function [111]. The gut microbiota appear to play a crucial role in migraine pathogenesis in both direct and indirect ways [116]. Direct evidence includes changes in gut microbiome composition, short-chain fatty acids, and cytokine release linked to migraine-like pain in animal models. Indirectly, probiotics, specific diets, and vagus nerve stimulation show that microbiome-based treatments might be effective for migraines [116].

Migraine in children often coexists with functional gastrointestinal disorders, underscoring the significant role of the gut–brain axis and gut microbiota in migraine development [121]. In a recent study, it was indicated that gut microbiota dysbiosis may contribute to the development of pediatric migraine [121]. The study identified a combination of seven gut bacterial genera with high diagnostic accuracy for pediatric migraine and found alterations in gut microbiota related to tryptophan metabolism. The kynurenic acid/quinolinic acid ratio emerged as a key diagnostic marker. These findings suggest that the gut microbiota, through the kynurenine pathway, may influence the pathogenesis of pediatric migraine, shedding light on the role of the gut–brain axis in shaping the onset and progression of migraines [121].

### 6.4. Role of Inflammatory Cytokines in Pediatric Migraine

Neurogenic neuroinflammation plays a crucial role in the pathophysiology of migraine including the transition from episodic migraine to chronic [122,123,124]. “Neurogenic inflammation” theory suggests that migraine pathogenesis is associated with the activation of meningeal afferents, neuropeptides, and vasodilation, resulting in inflammation and trigeminovascular system activation [124,125]. Inflammation triggers neuropeptide release and secretion of proinflammatory mediators, cytokines, free radicals, and kinins, which may offer migraine treatment avenues [125]. Cytokines are soluble signaling peptides primarily secreted by immune cells, acting as crucial mediators of immune response and inflammation [126]. Their involvement in migraine pathogenesis has gained widespread recognition among researchers [126]. It is well-recognized that pro-inflammatory cytokines like Interleukin-(IL)-1β and tumor necrosis factor (TNF)-α may increase in migraine patients during or between attacks [127]. A meta-analysis revealed that serum levels of CRP, IL-1β, IL-6, and TNF-α were elevated in migraine patients compared to healthy controls [128]. Preclinical research also shows that cytokines such as IL-1β and IL-6 can activate and sensitize meningeal and muscle nociceptors, suggesting their role in contributing to migraine pain [127].

A recent study found that cytokine levels, including IL-4, TNF-α, IL-17A, and IL-12p70, are elevated in children with migraine [126]. The study also revealed that higher levels of IL-12p70 and IL-17A are associated with an increased risk of pediatric migraine, highlighting their potential as predictive markers for the condition [126]. Another study reported that children with migraine showed significantly higher levels of pentraxin-3 (PTX-3), insulin, and insulin resistance, suggesting a role for inflammation and vascular endothelial dysfunction in pediatric migraine [129]. PTX-3 is a plasma protein involved in acute and chronic inflammation and vascular dysfunction [130]. Elevated PTX3 levels, correlated with migraine severity, suggest that ongoing inflammation and endothelial dysfunction contribute to pediatric migraine pathogenesis [129]. Likewise, a study explored the relationship between migraine and inflammation, finding higher C-reactive protein (CRP), creatinine, and thyroid-stimulating hormone levels, and lower serum iron levels in migraine patients [125]. Mean platelet volume was notably higher in girls with migraine, suggesting a possible link between inflammation, platelet activity, and migraine [125]. It is well-recognized that CGRP plays a significant role in pediatric migraine, particularly in its involvement in inflammation [131]. CGRP pathways are active from early development and are linked to elevated CGRP levels in children and adolescents with migraine. This link to inflammation supports the effectiveness of CGRP-targeting treatments like triptans in children and adolescents, though more research is needed for newer therapies like gepants and CGRP antibodies [131]. Furthermore, a recent study reported that CGRP and pituitary adenylate cyclase-activating polypeptide -38 levels are elevated in pediatric migraine, especially during the ictal phase and in those with aura. These peptides are crucial for diagnosing pediatric migraine, with their combination providing enhanced diagnostic accuracy [132]. In summary, these studies highlight the pivotal role of inflammation in pediatric migraine, suggesting that targeting inflammatory pathways could improve diagnosis and treatment strategies for young patients.

## 7. Therapeutic Strategies

Migraine treatment in children and adolescents should begin as early as possible after identifying early symptoms [61]. Previous studies suggest that the most effective treatment for pediatric migraines involves a biopsychosocial approach with interdisciplinary care, incorporating timely acute pharmacological interventions, patient education on self-management, and psychological therapies like biofeedback, relaxation, and cognitive–behavioral therapy [61,99,133]. The goal of managing an acute migraine is to achieve pain relief within 1–2 h, but this is not always possible [134]. Treatment should aim to shorten the episode, reduce pain, and alleviate symptoms like nausea, vomiting, dizziness, and sensitivity to light and noise [61]. The treatment regimen for pediatric migraines includes both non-pharmacologic and pharmacologic approaches. Non-pharmacologic methods involve sleep hygiene, dietary management, stress reduction, exercise, and trigger avoidance, while pharmacologic options include beta-blockers, calcium channel blockers, serotonin antagonists, antidepressants, and antiepileptics [99]. The following section focuses on non-pharmacological, pharmacological, and nutraceutical treatments, offering a comprehensive approach to managing pediatric migraines.

### 7.1. Non-Pharmacological Treatments

Non-pharmacologic therapies demonstrate effects similar to drug therapies for headache prevention while often being more accessible and less expensive [135]. Non-pharmacological treatments for pediatric migraines include lifestyle changes like better sleep, diet management, exercise, and stress reduction to reduce migraine frequency [136]. Behavioral therapies such as biofeedback, behavioral therapy, and mindfulness help with coping strategies, while non-invasive neuromodulation, like trigeminal and vagal nerve stimulators or transcranial magnetic stimulation, can reduce migraine severity through neural stimulation [136]. The subsequent sections specifically focus on lifestyle modifications and cognitive behavioral therapy (CBT) as key non-pharmacological strategies for pediatric migraine management.

#### 7.1.1. Lifestyle Modifications

Evidence-based guidelines for pediatric migraine prevention emphasize lifestyle assessments [137]. Educating patients about lifestyle factors is a key component of any effective headache management strategy [138]. Modifiable lifestyle factors include proper sleep hygiene, diet, exercise, and stress management reduce migraine severity and associated anxiety, while high caffeine intake, poor hydration, irregular meals, inactivity, smoking, and excessive alcohol use can trigger or prolong headaches [138]. The CHAMP study recommended several healthy habits for children and adolescents to prevent migraine attacks, including proper hydration, consistent physical activity, avoiding meal skipping, and ensuring a regular sleep schedule [139]. In the following sections, we discuss key lifestyle modifications for effective pediatric migraine management, covering stress reduction, dietary interventions, hydration, sleep hygiene, physical activity, and managing screen exposure.

##### Stress Management and Pediatric Migraine 

Stress has the strongest empirical link to the onset of migraine attacks among lifestyle factors [140]. Acute stress is a major trigger for migraines, with studies indicating it causes 50–80% of episodes in both adults and children [138]. Stress not only triggers migraine onset but also promotes its chronification, with higher frequency linked to increased perceived stress [136]. In children and adolescents, school-related stress from peer issues, teachers, and academic demands is a key trigger for headaches. Family background also plays a significant role, as factors like punitive parenting, family conflicts, and parental depression increase the risk of migraines [138]. Pediatric headache management should incorporate lifestyle improvements such as healthy dietary habits, good sleep hygiene, and regular physical activity. Additionally, integrating biopsychosocial techniques, including relaxation, biofeedback, and cognitive–behavioral therapies, is recommended to effectively reduce stress-related headaches and promote overall well-being in children and adolescents [141]. Caregivers should provide calm, supportive assistance without dismissing or overreacting to the child’s pain [61].

##### Dietary Interventions for Pediatric Migraine Management 

Diet plays a crucial role in managing migraines, yet no single dietary regimen is universally recommended for migraine sufferers [142]. The literature indicates that a specific dietary pattern, including weight loss, low-calorie diets, or fatty acid supplementation, prevents overweight and obesity, and is linked to improved migraine progression [143]. Ketogenic diets (KDs) are emerging as promising migraine therapies [144]. In adults, KDs reduce neuroinflammation and cortical spreading depression, showing significant benefits [136]. Although evidence in pediatric migraine is limited, case reports such as one involving a six-year-old with Glut1 deficiency syndrome and hemiplegic migraine suggest potential benefits. However, frequent side effects may limit adherence in children [136,145]. Additionally, certain foods may trigger migraines in pediatric populations, making dietary management essential for controlling symptoms. Food triggers, including chocolate, caffeine, alcohol, nitrites, aspartame, and gluten, may contribute to migraines in children, like their effect in adults. Avoiding these triggers may help prevent migraine episodes [136].

Irregular meal patterns in children and adolescents have also been linked to more frequent and severe migraine attacks [142,146]. In particular, skipping meals often triggers severe headaches with mild nausea, accompanied by early signs such as yawning, pallor, sweating, sweet cravings, and mood changes that mirror those of missed meals [138]. Migraineurs are advised to eat at regular intervals to sustain stable blood glucose levels and prevent the onset of migraine warning signs, such as yawning, fatigue, or mood swings [142]. Consequently, clinicians are advised to implement individualized dietary interventions for pediatric migraine management that emphasize consistent meal timing, exclusion of identified dietary triggers, and maintenance of optimal nutritional homeostasis.

##### Hydration in Pediatric Migraine 

Adequate hydration is crucial in pediatric migraine management [138]. While pediatric data are limited, adult studies suggest that increased water intake reduces migraine severity and frequency. Dehydration is common in adolescents, implying hydration may alleviate migraine. From a pathophysiological perspective, fluids may enhance blood volume, improve brain oxygen delivery, and help maintain proper plasma osmolality and sodium balance [138]. Additionally, headache specialists recommend improving hydration to prevent adverse effects associated with various abortive and preventive medications, as dehydration may exacerbate medication side effects in pediatric migraine patients [147]. Currently, adequate hydration with non-caffeinated fluids is recommended, typically around 80–100 oz daily for adolescents and approximately 48–64 oz per day for younger children [147].

##### Sleep Hygiene Practices for Pediatric Migraine 

Sleep is recognized as a natural remedy for migraines, and effective sleep hygiene can aid in managing chronic migraine [148]. Sleep hygiene programs promote a consistent schedule for sleep and waking, sufficient time in bed, avoidance of substances that may disrupt sleep, and a conducive sleep environment to improve sleep quality and potentially reduce the risk of headaches and migraine attacks [138]. The American Academy of Sleep Medicine (AASM) advises that children aged 6–12 years should sleep 9–12 h per 24 h, while teenagers aged 13–18 years should aim for 8–10 h within a 24-h period [149]. Insomnia and sleeping less than 8 h are key risk factors for headaches in children and adolescents [150]. The key guidelines of sleep hygiene include maintaining a quiet, dark, and cool bedroom, reserving the bed exclusively for sleep, avoiding electronic devices such as phones, tablets, or televisions in the sleeping area, and adhering to a consistent bedtime schedule [151].

##### Role of Physical Activity in Pediatric Migraine Management 

Physical activity, especially aerobic exercise, is a crucial lifestyle factor for headache management. It modulates pain through opioid, serotonin, and NMDA mechanisms in the rostral ventromedial medulla to promote analgesia while also alleviating stress, depression, and anxiety commonly linked to chronic pain [138]. Obesity and low physical activity are linked to higher migraine frequency in children and adolescents [136,152]. School-aged children with migraines favor sedentary activities and exercise less than peers, while adolescents with headaches also lead more sedentary lifestyles [153,154]. Approximately 50–60% of pediatric migraine patients are obese or overweight, with obesity increasing headache risk by 40% and leading to more frequent, severe attacks; therefore, promoting physical activity and weight loss may help reduce migraine frequency and severity [136]. A recent randomized trial in 51 pediatric migraine patients found that aerobic and neck exercises significantly reduced migraine frequency, severity, and duration. Notably, the aerobic exercise group experienced greater reductions in attack frequency, especially among those with neck pain [138]. Considering the above evidence, promoting aerobic exercise and weight management in pediatric migraine patients can significantly reduce attack frequency, severity, and duration.

##### Impact of Screen Time on Pediatric Migraine 

Over the past decade, the communication and information landscape has drastically evolved due to the rapid adoption of portable devices such as smartphones and tablets, providing constant internet access [155]. Research suggests a potential link between excessive screen exposure and pediatric headaches [156]. A recent cross-sectional study found that increased screen time is associated with higher headache frequency in children and adolescents diagnosed with migraine with aura [157]. Common devices contributing to screen time include smartphones, tablets, computers, televisions, and video gaming consoles. Screen time is typically categorized into school-related, involving online classes and digital homework, and leisure-related, involving personal entertainment, gaming, or social media activities [157]. A large epidemiological study found that 63% of adolescents (11–15 years) use mobile phones, with 23% experiencing migraines and 5% suffering from insomnia, a known migraine-related condition [158]. A retrospective study on 102 children and adolescents identified video game overuse as the fourth most common migraine trigger [27]. A comparative study found that children with migraines had significantly higher daily screen time (median 2.0 h) compared to those without migraines (median 1.0 h), highlighting screen exposure as a potential migraine risk factor [159]. Additionally, another study found an 80.6% prevalence of headaches in adolescents, linking excessive electronic device use to migraine development. The study classified headaches as 17.9% tension-type, 19.3% migraine, and 43.4% other types [160]. In light of the above evidence, pediatricians should recommend limiting recreational screen time, encouraging device-free periods, parental supervision, and restricting school-related screen use to a maximum of 2 h daily, with regular breaks (20 s every 20 min), to effectively reduce pediatric headaches [156].

#### 7.1.2. CBT in Pediatric Migraine

CBT is recognized as the gold standard psychological intervention for pediatric migraine management, effectively reducing headache frequency and associated disability [161]. CBT integrates cognitive and behavioral techniques to help patients manage environmental triggers, emotions, and thought patterns associated with headaches [162]. Furthermore, it serves as a valuable alternative to pharmacotherapy for reducing pediatric headache-related disability and has been shown to enhance the quality of life in children and adolescents [163]. Specifically, CBT for pediatric migraine includes family-oriented psychoeducation, identifying headache triggers through self-monitoring, teaching children effective pain-coping strategies, and encouraging the practical application of learned skills in everyday life [136].

Notably, a study indicated that preventive medications for pediatric headache may be largely effective due to a placebo effect. It found that CBT significantly reduces headache frequency and related disability in youth, while also offering a favorable benefit–risk profile with almost no negative side effects. Consequently, CBT should be considered a first-line treatment, either alone or in combination with medications, for effectively managing pediatric headache and migraine [164]. Supporting these findings, a randomized clinical trial demonstrated that combining CBT with amitriptyline significantly reduced headache frequency and disability in pediatric chronic migraine patients, with sustained improvements in 86–88% of participants at one-year follow-up, compared to headache education plus amitriptyline alone [165]. Likewise, another randomized clinical trial indicated that cognitive CBT combined with amitriptyline markedly reduced monthly headache frequency (≤4 days/month) in pediatric chronic migraine patients compared to headache education plus amitriptyline alone, with sustained improvements at one-year follow-up [166]. Furthermore, a systematic review and meta-analysis found that CBT significantly reduces migraine frequency and disability in pediatric patients, and effectively complements pharmacological treatments [167]. Considering the above evidence, CBT is strongly supported as a key component of pediatric migraine management, significantly reducing headache frequency and disability while enhancing quality of life. Clinicians should prioritize CBT in pediatric migraine treatment plans.

### 7.2. Pharmacological Treatment for Pediatric Migraine

The selection of medication depends on the child’s age, sex, pubertal stage, symptom recognition, comorbidities, migraine characteristics, and safety profile, with early medication administration key to better outcomes [61]. Before evaluating effectiveness, preventive treatment should be taken regularly for 6–8 weeks at the optimal dose. If successful, continue for 6–12 months before reassessing for withdrawal [61]. Although pain relief is the focus, managing symptoms like nausea or dizziness may be prioritized. Opioids are not recommended due to the risk of overuse headaches, and Dihydroergotamine is reserved for severe cases. Paracetamol or ibuprofen are common, but alternative routes like nasal or subcutaneous may be needed if nausea and vomiting occur [61]. The following subsections discuss the pharmacological agents used in pediatric migraine.

#### 7.2.1. Simple Analgesics

The primary intervention for acute migraine in children is prompt treatment with nonsteroidal anti-inflammatory drugs (NSAIDs) [133]. Paracetamol (acetaminophen) and ibuprofen are common over-the-counter treatments for pediatric migraine and have demonstrated efficacy and safety for acute management in children under 12 [168]. The initial paracetamol dose is 15–20 mg/kg, followed by 10 mg/kg every 4–6 h, while ibuprofen is given at 7.5–10 mg/kg. Meta-analyses show that ibuprofen is more effective than placebo in relieving pain within 2 h of migraine onset. In contrast, no significant difference between paracetamol and placebo was found in pain relief after 2 h [169,170]. Ibuprofen is believed to work by inhibiting prostaglandin synthesis and by enhancing central analgesic pathways, which strengthen descending pain modulation and reduce activity in brain structures that focus attention on pain [171]. Naproxen sodium is often the next NSAID used and has been shown effective in adolescents when combined with sumatriptan in a single pill [133]. Aspirin is effective for acute migraine treatment but is not recommended for children and adolescents due to the risk of Reye’s syndrome [172].

#### 7.2.2. Triptans

For pediatric patients who do not respond to NSAIDs for acute migraine relief, considering the use of a triptan, either alone or in combination, is recommended [133]. Triptans are currently the only FDA-approved migraine-specific treatments for acute care in children and adolescents [173]. Triptans are a group of selective serotonin (5-HT) 5-HT1B/1D receptor agonists, likely to relieve migraines by blocking ascending pain signals in the trigeminal nucleus caudalis and reducing the release of pro-inflammatory mediators at peripheral trigeminal nerve endings, despite their vasoconstrictive effects [30,173]. Over a dozen randomized controlled trials have evaluated the efficacy and safety of triptans versus placebo for treating pediatric migraines [169]. Rizatriptan is the only triptan approved for children aged 6–17, with no migraine-specific treatments available for those under 6 [173]. Liquid intranasal formulations, including sumatriptan, zolmitriptan, and almotriptan, are the most studied in children [174]. Pharmacokinetic studies indicate that triptan intra-nasal drug delivery has higher bioavailability and a quicker onset of action compared to oral forms [175]. The European Medicine Agency approves Sumatriptan nasal sprays, while zolmitriptan, almotriptan, and rizatriptan nasal sprays are FDA-approved for patients aged 12 and older [174]. Triptans should be used cautiously for frequent migraines to avoid medication overuse headache [169]. They are not recommended for children with hemiplegic migraine or complex aura due to the theoretical risk of cerebral vasoconstriction, though no evidence suggests significant clinical effects [61].

#### 7.2.3. Ergot-Based Therapy

Ergot alkaloids like ergotamine have been used since the 1970s for acute migraine treatment [176]. Dihydroergotamine (DHE) acts on serotonin, dopamine, and adrenergic receptors, blocking nociceptive pathways in the trigeminal vascular system via 5-HT1D agonism and causing vasoconstriction through 5-HT1B receptors [177]. Although placebo-controlled studies for DHE are limited, evidence supports its tolerability, dosing, and efficacy in pediatric patients with intractable migraine [178]. However, responses to DHE in pediatric status migrainosus may vary depending on the migraine subtype [179].

#### 7.2.4. Topiramate

Topiramate, an antiepileptic drug commonly used for migraine prevention in adults, was the first FDA-approved preventive treatment for migraines in adolescents aged 12 to 18 [180]. Topiramate primarily reduces neuronal excitation and enhances neuronal inhibition [181]. The ideal dosage of topiramate ranges from 1 to 2 mg/kg per day, with a maximum daily dose of 200 mg [139]. However, the Childhood and Adolescent Migraine Prevention (CHAMP) trial, the largest study to date comparing migraine prevention medications in youth, was discontinued early when interim findings revealed no significant difference between topiramate and placebo in reducing headache days [182,183]. Despite topiramate’s effectiveness in reducing headache frequency in some youths, it carries numerous side effects. Children should be closely monitored for potential adverse reactions, including appetite loss, weight loss, fatigue, dry mouth, memory, and cognitive impairment [139].

#### 7.2.5. Valproic Acid

Sodium valproate enhances gamma-aminobutyric acid activity in the brain and may inhibit cortical neuronal hyperexcitability [184]. Valproic acid plays a significant role in migraine prevention in adults, with several studies supporting its effectiveness in pediatric migraine [185]. However, its teratogenic (class X) and ovarian effects restrict its use in females [186]. A pediatric study showed that intravenous (IV) valproic acid was well-tolerated, with over 80% of children aged 10 and older maintaining serum levels of 80–100 mcg/mL, with nausea being the most common side effect [187]. Likewise, an IV valproic acid bolus followed by a 24–48 h continuous infusion has been suggested as an effective treatment for children with status migrainous [188].

#### 7.2.6. Levetiracetam

Levetiracetam, an antiepileptic with neuromodulatory properties, is commonly used to prevent migraines in adults but has limited research supporting its use in children [189]. Preliminary studies indicate that levetiracetam significantly reduces migraine frequency and disability in pediatric patients, with 83% showing improvement, while adverse reactions, mainly irritability, were reported in 18% of cases [189,190]. In an open-label study of 30 children aged 6–19, 56% found a >50% reduction in headaches after 10 weeks, though 30% dropped out due to noncompliance, side effects, or lack of efficacy [190]. Future research should focus on large, well-designed RCTs to better assess the efficacy and safety of levetiracetam for pediatric migraine prevention.

#### 7.2.7. Amitriptyline

Amitriptyline, a tricyclic antidepressant, has proven effective in migraine pediatric prevention [191,192]. Amitriptyline’s migraine prevention likely involves serotonin and noradrenaline reuptake inhibition, enhancing serotonergic transmission and activating α2-adrenoreceptors for pain relief [193]. It also affects multiple systems, including anticholinergic, antihistaminergic actions, and modulation of ion channels and various receptors like adrenergic α1, NMDA, and opioid receptors [193]. The CHAMP study found it as effective as a placebo in reducing migraine days by over 50%, but its efficacy improves when combined with cognitive behavior therapy [139,165]. In a trial comparing amitriptyline and cinnarizine for pediatric migraine, amitriptyline showed faster, longer-lasting effects in reducing headache frequency and duration, with no serious adverse events [191]. Amitriptyline is typically prescribed at a starting dose of 0.25–1.0 mg/kg/day, administered usually in the evening, with gradual dose escalation to minimize side effects like drowsiness, increased appetite, weight gain, dry mouth, and behavioral changes [61].

#### 7.2.8. Cyproheptadine

Cyproheptadine, a first-generation antihistamine, has been used to prevent migraines in young children since the 1980s [147]. Although it can cause sedation and increased appetite, these side effects typically do not interfere with daily life in this age group [194]. While it is less effective than other treatments, its safety profile makes it a suitable option, particularly showing more effectiveness in chronic migraine patients [195].

#### 7.2.9. Cinnarizine

Cinnarizine has been demonstrated to be a safe and effective treatment for reducing the frequency, intensity, and duration of headaches in children [191]. Cinnarizine, an antihistamine and calcium channel blocker, may prevent migraines by inhibiting cerebral vascular smooth muscle, blocking calcium channels to raise the excitatory threshold, and reducing neuroinflammation like CGRP release [61]. A previous study comparing the prophylactic efficacy of cinnarizine and placebo in pediatric migraine found both effective in reducing attack frequency, severity, and duration in children; however, cinnarizine proved significantly better at reducing headache frequency [196].

#### 7.2.10. Propranolol

Propranolol is a non-selective beta-blocker with good blood–brain permeability [147]. It is widely accepted for migraine prevention, though evidence in children is limited, with some reports suggesting it may reduce headache frequency by at least 50% compared to placebo [174]. Propranolol is generally safe, though side effects like bradycardia and hypotension may occur, requiring blood pressure monitoring. It should be avoided in asthma patients [61].

#### 7.2.11. Emerging Pharmacological Treatments

Recent advancements in migraine therapy have introduced novel pharmacological options, with several undergoing clinical trials, to expand treatment possibilities for pediatric patients [197,198]. Monoclonal antibodies targeting CGRP and its receptor, including erenumab, fremanezumab, galcanezumab, and eptinezumab, along with CGRP receptor antagonists (gepants) such as rimegepant, ubrogepant, atogepant, and zavegepant, have been developed as effective acute and preventive treatments for migraine [199]. These therapies, including both monoclonal antibodies and CGRP receptor antagonists, are now undergoing evaluation in pediatric clinical trials to assess their safety and efficacy, emphasizing the need for expanded treatment options for children and adolescents with migraine [199].

As reported in April 2024, 16 clinical trials were underway to evaluate the safety and efficacy of CGRP inhibitors in pediatric patients, with results expected in the coming years [198]. The pediatric and adolescent headache special interest group of the AHS recommends considering anti-CGRP monoclonal antibodies for postpubertal pediatric patients with at least eight headache days per month, a PedMIDAS score of ≥30, and failure of two or more preventive treatments, including medications, cognitive behavioral therapy, natural supplements, or neuromodulation devices [200].

Additionally, lasmiditan, a selective serotonin (5-HT1F) receptor agonist approved for acute migraine treatment in adults, demonstrated favorable pharmacokinetics and tolerability in pediatric patients aged 6 to <18 years. The Phase I trial supports weight-based dosing, with safety profiles comparable to adults and no new safety concerns, warranting further investigation for pediatric migraine management [201].

### 7.3. Nutraceuticals in Pediatric Migraine

Nutraceuticals, as defined by the American Nutraceutical Association, are foods or products with health-enhancing properties, from dietary supplements to genetically modified foods [202]. Due to limited high-quality efficacy data on pharmacologic migraine prevention in children and adolescents, nutraceuticals have become a common alternative treatment for managing headaches in this population [203]. Although nutraceuticals are generally considered safe, it is a misconception to assume they are entirely free from significant side effects [204]. The following section discusses nutraceuticals in detail, including riboflavin, vitamin D, coenzyme Q10, butterbur, magnesium, polyunsaturated fatty acids, melatonin, and Ginkgolide B, highlighting their potential benefits and associated risks in managing pediatric migraine.

#### 7.3.1. Riboflavin

Riboflavin (vitamin B2) is a precursor to flavin mononucleotide and flavin adenine dinucleotide [136]. It regulates vascular and neuronal functions, inhibits neuroinflammation, and helps repair mitochondrial dysfunction linked to migraine [136,205]. A prophylactic dose of 50–400 mg/day is recommended for school-age children to reduce the frequency and duration of migraine attacks [136]. Due to conflicting results and limited evidence, the efficacy of riboflavin for pediatric migraine prevention and predictors of treatment response remains unclear [206].

#### 7.3.2. Vitamin D

Vitamin D acts as a neurosteroid, supporting brain development, and synaptic plasticity, and functions as an immunomodulator [207]. Vitamin D prevents neuroinflammation linked to migraine, musculoskeletal pain, and other headaches by inhibiting prostaglandin E2, and nitric oxide production, and reducing oxidative stress, a key migraine trigger [208]. Multiple studies have shown a strong link between vitamin D deficiency and primary headaches in children [209,210]. A randomized trial showed that combining topiramate with vitamin D3 was more effective than topiramate alone in reducing headache frequency and improving daily function in children with migraines [211]. Similarly, in a non-randomized study of 37 children and adolescents with migraines or tension headaches, vitamin D levels were measured, and supplementation was given based on initial levels. After 3 months, a reduction in headache intensity was observed, with follow-ups at 3 and 6 months [203].

#### 7.3.3. Coenzyme Q10

Coenzyme Q10 (CoQ10) is the third most popular supplement after fish oil and multivitamins. Known for its antioxidant properties and role in mitochondrial bioenergetics, it is also considered a potential treatment for diseases involving oxidative stress [212]. Its use is supported by evidence of mitochondrial dysfunction in migraine patients [204]. A randomized controlled trial of 62 pediatric patients compared coenzyme Q10 (100 mg/day for 4 months) with placebo for migraine reduction, showing a decrease in attack frequency and intensity in both groups without statistical significance and no significant adverse events [213]. In a study of 72 patients aged 5–15, only one reported abdominal discomfort after CoQ10 supplementation. Based on these findings, CoQ10 was recommended as a potential prophylactic treatment for migraines [214].

#### 7.3.4. Butterbur (Petasites Hybridus)

Butterbur, a plant from the Asteraceae family, is traditionally used to treat migraines, tension headaches, stomach pain, allergic rhinitis, asthma, and gastrointestinal spasms [215,216,217]. *Petasides hybridus* extracts have been shown to inhibit cyclooxygenase-2, reduce inflammation by blocking leukotriene production, and inhibit L-type voltage-gated calcium channels, limiting intracellular calcium accumulation [218]. Preliminary studies indicate butterbur may be effective preventing migraines in children and adolescents [219,220]. Due to its hepatotoxicity, butterbur is available in various formulations. Petadolex^®^, a butterbur formulation, was studied in children and found to be less hepatotoxic [221]. Due to conflicting reports, butterbur is generally not recommended for treating migraines in children and adolescents [183].

#### 7.3.5. Magnesium

Magnesium (Mg), a key intracellular ion, plays a vital role in biological processes such as oxidative phosphorylation, energy production, glycolysis, and protein and nucleic acid synthesis [222]. Magnesium deficiency is linked to migraine pathogenesis by altering neurotransmitter secretion, triggering CSD, and increasing platelet aggregation [223]. Magnesium mitigates both vascular and neurogenic aspects of migraines by blocking NMDA receptors, which moderates the release of substance P and regulates nitric oxide production, thereby helping to control migraine symptoms [224,225]. In a pediatric randomized controlled study, 118 children and adolescents with migraines were treated with magnesium oxide (9 mg/kg) versus placebo, with no significant differences observed in primary outcomes [226]. However, children and adolescents on magnesium (400 mg daily) for migraine prevention in another study showed reduced pain intensity when treated with acetaminophen or ibuprofen, compared to those not on magnesium therapy [227].

#### 7.3.6. Polyunsaturated Fatty Acids

Polyunsaturated fatty acids (PUFAs) are key components of cell membranes and are important nutrients for treating non-alcoholic fatty liver, autoimmune disorders, and various chronic diseases [228]. Among PUFAs, Omega-3 is the most significant, offering antioxidant, anti-inflammatory, and neuroprotective benefits [229]. Studies have assessed the effectiveness of PUFAs in preventing migraines in children and adolescents [179,203]. In a double-blind study, 25 children with migraines were treated for 2 months with sodium valproate (20 mg/kg daily) combined with either a fish oil compound (eicosapentaenoic acid and docosahexaenoic acid) or a placebo. Both groups showed significant reductions in headache frequency and pediatric migraine disability assessment score (PedMIDAS), but no statistically significant differences were found between the groups [230].

#### 7.3.7. Melatonin

Melatonin is a hormone naturally produced by the pineal gland in the brain, playing a crucial role in regulating the sleep–wake cycle (circadian rhythm) [231]. Melatonin levels might be reduced in migraine, contributing to its pathogenesis, and it has been used as an effective treatment due to its anti-inflammatory, analgesic, antioxidant properties, neurovascular modulation, and protection against glutamate neurotoxicity [232]. A quasi-experimental study reported significant reductions in headache frequency, severity, and duration in 60 children treated with melatonin (0.3 mg/kg) for three months [233]. A single-blinded RCT compared melatonin (0.3 mg/kg) and amitriptyline (1 mg/kg) for pediatric migraine prevention in 80 children. Amitriptyline was more effective, but melatonin had fewer side effects, making it a safer option for migraine prophylaxis [232].

#### 7.3.8. Ginkgolide B

Ginkgolide B, an extract from Ginkgo biloba leaves, modulates glutamate action in the central nervous system [234]. Open-label studies suggest that it may be effective for migraine prevention in children, showing favorable outcomes with no reported adverse events when used alongside other complementary treatments [234].

## 8. Follow-Up and Prognosis of Pediatric Migraine

Long-term studies indicate that pediatric migraines persist into adulthood, often transforming their characteristics and classifications. A 30-year follow-up study found that while 29% of patients achieved complete remission, 71% continued to experience headaches, with fluctuations between migraine and TTH over time [235]. Despite persistent symptoms, most patients managed their headaches with nonprescription analgesics, relaxation techniques, and trigger avoidance, highlighting the importance of long-term self-management strategies [235]. Similarly, a 25-year follow-up study showed that although 33% of children with migraines achieved remission, 67% continued to experience headaches, with most reporting improvement [236]. Male sex was associated with a more favorable prognosis, whereas female patients were more likely to experience persistent headaches, potentially due to hormonal influences. The study also found that some patients transitioned between migraine and TTH, reinforcing the need for long-term monitoring and individualized management approaches [236].

A 20-year follow-up study further emphasized the evolving nature of pediatric headaches, revealing that while 27% of childhood headache patients achieved remission, 73% continued to experience headaches, often shifting from one type to another [237]. TTH patients had a higher likelihood of remission compared to migraine patients, and initial headache severity was a predictor of long-term persistence. Stress emerged as an increasingly recognized trigger, and most patients relied on nonprescription medications or non-pharmacological strategies for symptom control [237]. Likewise, a 10-year follow-up study comparing pediatric migraine and TTH found that while 81% of migraine patients showed improvement, only 24% achieved complete remission, whereas 45% of TTH patients became headache-free [238]. The likelihood of retaining the initial diagnosis was greater for migraine (59%) than for TTH (37%), and 16–18% of patients experienced a diagnostic shift between the two. The presence of aura and photophobia significantly predicted migraine persistence, reinforcing the need for tailored treatment approaches [238].

While long-term studies focus on headache evolution over decades, short-term prognosis is equally relevant in clinical practice. A study analyzing 13,160 visit pairs from 5316 pediatric migraine patients found that the majority improved with multimodal care, with 56.8% having fewer headaches, and 34.8% experiencing a ≥50% reduction [239]. However, 14.5% of patients worsened, with risk factors including older age, female sex, chronic migraine, status migrainosus, depressive symptoms, and high disability scores. In contrast, summer visits, caffeine consumption, and pharmaceutical treatments were associated with better outcomes [239]. These findings highlight the need for early identification of high-risk patients to guide targeted interventions and optimize management strategies.

Overall, TTH generally has a more favorable prognosis than migraine, with sex, headache severity, and psychosocial factors influencing outcomes. Effective management requires a personalized, long-term approach integrating pharmacological and non-pharmacological strategies, focusing on patient education, trigger avoidance, and lifestyle modifications.

## 9. Conclusions

Pediatric migraine is a neurological disorder with a multifactorial etiology, encompassing genetic, environmental, neuroinflammatory, and other biopsychosocial factors, significantly impacting children’s quality of life, academic performance, and social interactions. Accurate diagnosis and timely treatment are essential for preventing long-term disability and improving the quality of life in affected children. This review explores the diagnostic criteria, sex differences, triggers, clinical features, comorbidities, and underlying mechanisms of pediatric migraine. Additionally, it evaluates current treatment approaches, encompassing pharmacological agents, non-pharmacological therapies, and nutraceuticals. Emerging pharmacological therapies, such as CGRP inhibitors and serotonin receptor agonists, offer promising new treatment avenues, while lifestyle modifications, cognitive behavioral therapy, and patient education play a pivotal role in migraine prevention and management. A comprehensive, multidisciplinary approach is essential for optimizing diagnosis and treatment, preventing progression to chronic migraine, and improving outcomes for pediatric patients. Future research should focus on individualized treatment strategies, novel therapeutic targets, and long-term follow-up studies to improve clinical outcomes. By tailoring diagnostic and treatment strategies and emphasizing patient education on the multifactorial aspects of pediatric migraines, clinicians can enhance therapeutic outcomes, improve quality of life, and optimize long-term prognosis through a personalized, multidisciplinary approach.

## Figures and Tables

**Table 1:** Clinical differences between adult and pediatric migraine.

Criteria	Adult Migraine	Pediatric Migraine
Number of attacks	At least 5	At least 5
Headache Duration	4 to 72 h	1–72 h
Pain Location	Unilateral	Often bilateral or unilateral
Pain Quality	Pulsatile (Throbbing)	Pulsatile or constrictive
Pain Intensity	Moderate to severe	Moderate to severe
Aggravation by Activity	Yes	Yes
Associated Symptoms	Nausea, vomiting, photophobia, phonophobia	Nausea, vomiting, photophobia, phonophobia (often inferred)
Gastrointestinal Symptoms	Less frequent	More frequent (includes abdominal pain, diarrhea, constipation)

**Table 2:** Aura types in pediatric migraine: key symptoms and pathophysiological mechanisms.

Aura Type	Description	Key Symptoms	Pathophysiology
Typical Aura	Neurological symptoms lasting 5–60 min, followed by or occurring with headache.	Visual disturbances, sensory symptoms, speech deficits	Cortical spreading depression (CSD)
Brainstem Aura	Aura with reversible brainstem symptoms, common in pediatric migraine.	Vertigo, tinnitus, diplopia, altered consciousness	CSD, vasomotor dysfunction, hypothalamic involvement
Hemiplegic Migraine	Aura with motor weakness, often accompanied by visual, sensory, or speech aura.	Hemiparesis, visual, sensory, and speech deficits	Gene mutations (CACNA1A, ATP1A2, SCN1A), CSD
Retinal Migraine	Rare, involves monocular visual disturbances.	Monocular scotomata, scintillations, transient blindness	Retinal/optic nerve pathway disturbance

**Table 3:** Migraine phases in children: pathophysiological mechanisms and key clinical manifestations.

Phase	Description	Key Symptoms	Pathophysiological Mechanism
Prodrome	Occurs up to 48 h before the headache/aura.	Mood changes, fatigue, food cravings, photophobia, phonophobia, cranial autonomic signs	Hypothalamus activation, neurotransmitter changes
Aura	Lasts 5–60 min, usually preceding or occurring with the headache.	Visual disturbances, sensory and motor deficits, speech issues, brain stem symptoms	Cortical spreading depression
Headache	Throbbing/pounding, often frontal-temporal and bilateral.	Photophobia, phonophobia, nausea, vomiting, periorbital or neck pain	Trigeminovascular system activation
Postdrome	Follows headache, lasting up to 2 days or more.	Fatigue, mood changes, neck stiffness, irritability, difficulty concentrating	Locus coeruleus activation, reduced brain activity

**Table 4:** Overview of key pediatric episodic syndromes associated with migraine.

Episodic Syndrome	ICHD-3 Criteria	Associated Features	Onset	Treatment
Cyclic Vomiting Syndrome	At least 5 episodes of intense vomiting, with periods of symptom-free intervals.	Vomiting, light/noise sensitivity	5 years	Sumatriptan, Antiemetics, Lorazepam, Amitriptyline, Propranolol.
Abdominal Migraine	At least 5 attacks of abdominal pain with nausea, vomiting, and pallor lasting 2–72 h.	Pallor, anorexia, nausea, vomiting, family history of migraine.	5–9 years	Rest, Hydration, Analgesics, Pizotifen, Amitriptyline.
Benign Paroxysmal Vertigo	At least 5 episodes of vertigo, with nystagmus, vomiting, or ataxia.	Nystagmus, dizziness, and fearfulness.	3–4 years	Reassurance Pizotifen, Flunarizine (prolonged cases)
Benign Paroxysmal Torticollis	Recurrent head tilting with pallor, vomiting, or irritability.	Vomiting, lethargy, family history of migraine/colic, transient delay.	2–3 years	Supportive, Analgesics, Antiemetics, Potential Migraine prophylactics.
Infantile Colic	Recurrent episodes of crying, irritability, and fussing in infants under 5 months, with no other cause.	Maternal stress, normal growth, likely abdominal pain.	Birth to 5 months	Soothing strategies, no strong evidence for medications.
